# Empowering international students at Iran University of Medical Sciences: challenges, solutions, and opportunities

**DOI:** 10.1186/s12909-026-09493-0

**Published:** 2026-05-28

**Authors:** Rafat Bagherzadeh, Afsaneh Dehnad, Leila Nemati-Anaraki, Motahareh Aghashahi, Shiva Malgard

**Affiliations:** 1 English Language Department, School of Health Management and Information Sciences, Iran University of Medical Sciences, Tehran, Iran; 2 Health Management and Economics Research Center, Health Management Research Institute, Iran University of Medical Sciences, Tehran, Iran; 3 Department of Medical Library and Information Science, School of Health Management and Information Sciences, Iran University of Medical Sciences, Tehran, Iran; 4 Independent Researcher , Iran University of Medical Sciences, Tehran, Iran

**Keywords:** International students, Medical education, Higher education, Student challenges, Cultural adaptation, Iran

## Abstract

**Background:**

Internationalization in higher education offers significant benefits for universities and host societies, yet international students frequently encounter multiple adaptation challenges. This study explored the key challenges, potential solutions, opportunities, and future prospects for international students at Iran University of Medical Sciences (IUMS).

**Methods:**

A qualitative study was conducted in 2024 with 31 international students meeting defined inclusion criteria. Data were collected using semi-structured interviews in Persian or English, transcribed verbatim, and analyzed thematically using MAXQDA 2022. Trustworthiness was ensured through credibility, dependability, confirmability, and transferability criteria.

**Results:**

Four main categories emerged: (1) challenges — including language barriers, environmental adaptation, academic overload, cultural differences, nationality-based discrimination, personal isolation, and financial constraints; (2) solutions — such as curriculum restructuring, enhanced language courses, cultural awareness programmes, improved faculty English proficiency, and institutional advisory support; (3) opportunities — encompassing high-quality educational resources, diverse institutional support services, and personal skill development; and (4) future prospects — with most students intending to return to their home countries after graduation.

**Conclusion:**

International students at IUMS face interconnected linguistic, social, educational, cultural, and economic challenges that can adversely impact academic performance and well-being. Practical, multi-level interventions are necessary to support their integration and success. The findings provide actionable recommendations for university administrators and policymakers to improve the recruitment, retention, and satisfaction of international students and to strengthen the internationalization of medical education in Iran.

## Introduction

The rapid growth of information and communication technology has facilitated globalization, resulting in the widespread adoption of internationalization [1]. Internationalization in higher education is a global phenomenon [2], characterized by incorporating international, intercultural, or global characteristics into educational institution’s objectives, functions, or strategic planning [3, 4]. One strategy that numerous countries, especially developing nations, have employed to enhance the quality of their institutions is the establishment of global connections [5], along with the improvement of international student admission rules, as a major step towards adoption of internationalization [4]. International students are known as those who travel and stay abroad for educational purposes. With numbers rising in many nations, these students constitute a sizable portion of higher education systems around the world [6]. Regarding the continued increase in the international student population [7], recent projections and global reports indicate that the number of internationally mobile students has reached approximately 7 million worldwide BY 2025 [8–10].

Several factors contribute to the inclination of many countries to move towards globalization in higher education. These include the need to ensure a skilled workforce [7, 11], enhance their universities’ international presence and rankings [3, 7], upgrade academic institutions, expand educational opportunities, foster more competitive environments [4], and strengthen scientific and cultural connections [7]. To achieve these goals, these countries seek to attract more international students by improving the quality of educational programs and offering better services [10]. The admission of international students not only boosts revenue for higher education [12] but also fosters the growth of local businesses and stimulates the tourism industry [13].

In addition, these students can enrich the intellectual capital of their host country by introducing a diverse range of knowledge and skills from various disciplines, which can foster innovation, enhance research productivity, and contribute to the development of cutting-edge academic programs [4].

Internationalization in higher education can contribute significantly to cultural and ethnic diversity [14], which is crucial for facilitating connections and reducing conflicts between countries [10] by introducing different cultures to the host country. This fosters a diverse cultural community and enriches the culture and heritage of the host nation [15]. Therefore, supporting the emotional well-being of these students is as vital as facilitating their arrival at the destination university [7].

Moving to a new country presents students with various challenges and difficulties associated with living and studying in an unfamiliar environment [15–18]. For instance, international students must adapt to a new environment with cultural differences, language barriers, a different educational system, homesickness, weak social connections, and everyday challenges. Additionally, feeling of unhappiness and anxiety, loneliness, culture shock, financial issues, and racial discrimination can harm students’ performance, well-being, and health [19]. Consequently, students’ mental health and emotional state can be significantly influenced by the hospitable environment of universities, and institutions that exclusively concentrate on educational requirements may overlook critical factors that contribute to students’ success and well-being [20]. Thus, it is imperative to investigate the relevant policies, available support services, and effective strategies that can help these students overcome the obstacles and mitigate the challenges. Although Iran has emerged as a dynamic market for international education over the past decade, its reach remains largely regional, with 75% of students coming from Afghanistan and 25% from Iraq [7].

While a substantial body of research has examined the challenges faced by international students worldwide and proposed strategies to address these issues [10, 15, 16, 18, 19], other studies have focused on student satisfaction and the factors influencing their academic experiences across different countries, including Iran [1, 7, 21, 22]. In the Iranian context, Tajvar et al. (2024) explored the general challenges experienced by international students; however, their study did not specifically address the context of medical universities, which are characterized by distinct educational structures and clinical training demands.

Furthermore, existing studies have often focused on isolated aspects such as challenges or satisfaction, with limited attention to providing a comprehensive perspective that integrates challenges, potential solutions, and future opportunities. Therefore, this study aims to investigate the challenges faced by international students in a medical university in Iran, while also identifying practical strategies to address these challenges and exploring future opportunities in this context. The findings of this study can contribute to improving educational, research, and support services, thereby enhancing international student recruitment and advancing the internationalization of medical education in Iran.

## Methods

This qualitative study was conducted at IUMS in 2024. A qualitative research design was chosen to gain an in-depth understanding of international students’ lived experiences, perceptions, and challenges within the academic and sociocultural context of Iran University of Medical Sciences. Given the exploratory nature of the study and the limited prior qualitative research focusing on international students in Iranian medical universities, a qualitative approach was considered the most appropriate method to capture rich and contextualized data. The participants comprised 31 international students who voluntarily participated in the study. Students enrolled in the pre-clinical (basic sciences) phase were included in the study. Students in the clinical phase were excluded because a considerable proportion temporarily return to their home countries after completing theoretical training, making them less accessible for participation. Iranian students who had been admitted to the international campus through national university entrance examination were excluded from the study. Notification and invitations to participate in the interviews were sent through text messages to students by the International Education Department, as well as through announcements made by the researchers in the central library, a common study space for students, to further encourage participation. However, recruitment through an institutional channel may have introduced a potential selection bias, as students with negative experiences might have been less willing to participate.

A semi-structured interview guide served as the primary data collection instrument. Semi-structured interviews were considered appropriate because they allowed flexibility in exploring participants’ experiences while ensuring consistency across interviews, enabling both predefined topics and emerging issues to be addressed in depth. The interview guide was adapted from a previously published study and modified to fit the objectives of the present study. It included open-ended questions focusing on international students’ academic, social, and cultural challenges. The interviews were conducted using a semi-structured format, with predefined guiding questions while allowing flexibility to explore emerging topics, and adhered to pre-, during-, and post-interview procedures to ensure methodological rigor. Participants were thoroughly briefed on the study’s objectives, and their consent for audio recording was obtained. Open-ended questions were asked to establish rapport and trust with the participants. Face to face interviews lasting 25–40 min were held at the campus of the university where the students attended their classes or at the office of the researchers or based on their communication preferences, e-mail interviewing were also used. The questions were asked in English or in Persian if necessary and the participants were free to choose the language they would prefer to answer the questions since all international students participate in preparatory Persian conversation classes, which facilitate easier communication in Persian, particularly for those students from Afghanistan.

During the interviews, mutual trust was established between the researcher and each participant. Individual constructions were elicited and refined by interactions between the researcher and respondents [23]. According to Charmaz [24], “Qualitative interviewing provides an open-ended, in-depth exploration of an aspect of life about which the interviewee has substantial experience, often combined with considerable insights”. All taped sessions were transcribed to provide a written record of the interviews. The sample size was determined based on the principle of data saturation. Data analysis involved an interpretive process, and efforts were made to ensure rigor through careful coding, peer discussion, and maintaining an audit trail. These transcriptions or “field-notes” as suggested by Gay, Mills, and Airasian [25] were reviewed against the actual audio-tape for accuracy.

Data collection continued until saturation was achieved, which occurred after approximately 31 interviews, when no new themes or significant insights emerged. To ensure participant confidentiality, each interview sample was assigned a unique numerical code in sequential order. The trustworthiness of this study was supported by four criteria: credibility, dependability, confirmability, and transferability [26]. Data analysis followed a thematic approach using MAXQDA software (version 2022). Interview transcripts were conceptually coded, categorized, and reported across relevant dimensions and domains.

## Results

The research findings consistent with the research objectives are presented in four categories as follows: (1) challenges faced by international students at IUMS, (2) solutions to overcome challenges, (3) opportunities, and (4) future prospects for enhancing international education at IUMS. The categories and themes presented in the Results section were generated inductively through systematic coding and analysis of the interview transcripts. No pre-existing theoretical framework was imposed during data analysis. Instead, themes emerged directly from participants’ narratives through an iterative process of coding, categorization, and abstraction.

## Challenges faced by international students at IUMS

Participants reported various barriers and challenges affecting their academic performance. These challenges have been categorized and are presented in seven main categories, as detailed in Table 1: *language-related problems*, *environmental issues*, *educational challenges*, *cultural differences*, *national attitudes*, *personal problems*, *and financial issues*.

**Table 1:** Challenges Faced by International Students

Main Category	Subcategory	Concept
Language-Related Problems	Inadequate Listening & Speaking Skills in English & Persian	The insufficient proficiency of international students in English.
		The insufficient proficiency of international students in Persian.
		The insufficient proficiency of Iranian students in English.
	Lack of Reading Comprehension Skills in English & Persian	The lack of proficiency of international students in understanding English.
Environmental Issues	Disparity in Social Lifestyle	Dissatisfaction with the social lifestyle and inability to adapt to it in Iran
Educational Challenges	Lack of Structured Educational Programs for International Students	Overload of Academic Content
		Inadequate English Proficiency of Professors
		Differences in the educational system, regulations, and teaching methods
Cultural Differences	Inappropriate Behavior & Cultural Regulations at University	Disciplinary strictness towards international students
	Lack of Acceptance in Society	Discriminatory behavior of some citizens and professors towards non-Iranian nationalities
National Attitudes	Discrimination in the Enforcement of Disciplinary Rules	Discrimination in some disciplinary regulations between Iranian and international students
	Discriminatory & Unjust Behavior	Feeling discomfort due to the treatment of non-Iranian nationalities
Personal Issues	Lack of Family Communication	Homesickness and alienation
Financial issues	Monetary Problems	Living Costs
	Lack of Basic Banking Facilities	Difficulties in Transferring Funds & Currency Exchange

### Language-related problems

Language barriers were identified as a major concern reported by participants. The results indicate that international students encounter difficulties in comprehending academic material and engaging in academic discussions because of their limited proficiency in Persian and English. Specifically, the limited English language skills of Iranian students further complicate academic communication. The language-related challenges can be divided into two main categories: *insufficient listening and speaking skills in both English and Persian*, and *deficiencies in reading comprehension in these languages*.

Many students reported that both international and Iranian students struggle with English proficiency, which poses challenges in academic interactions and hinders their comprehension of course materials:

*“I’ll give an example about my university friends. They faced a big problem with language because they could only speak Arabic*,* and they couldn’t speak Persian or English.”* (Interview 4).

Additionally, many international students indicated that their limited proficiency in Persian posed challenges during academic and professional interactions:

*“In Iraq*,* most doctors speak Arabic*,* and it’s super easy to communicate with them but in Iran*,* most doctors speak Persian.”* (Interview 1).

Lack of reading comprehension skills hinders international students’ ability to understand academic texts. Some students expressed:

*“Reading and understanding books is hard from both perspectives. Since we’re new students*,* how can we read a book like ‘Junqueira’s Basic Histology’? It’s a huge histology book with difficult medical terms. So*,* we’re just starting out*,* our English isn’t that good*,* and because of all that*,* we’re facing a very*,* very*,* very difficult problem in this area.”* (Interview 1).

Furthermore, some students anticipated that their lack of Persian proficiency would create difficulties in communicating with Persian-speaking patients during potential clinical training. Although most international students at IUMS return to their home countries upon completing pre-clinical studies, they expressed concern that inadequate Persian language preparation could hinder the clinical experiences they might undertake elsewhere:

*“In Iran*,* we only have one semester of Persian*,* but as medical students*,* we need to speak much better Persian. We need to be fluent by the second or third year when we work with patients.”* (Interview 10).

The participants’ statements indicate that language barriers affected both daily communication and academic learning, particularly in clinical and classroom settings where Persian was predominantly used.

### Environmental issues

A disparity in social lifestyles was reported as one of the environmental challenges faced by international students at IUMS. The analysis of interviews revealed that, in addition to other factors directly impacting this challenge, specific conditions and characteristics may also contribute to the emergence or persistence of these challenges. A notable challenge identified is the dissatisfaction with the social lifestyle in Iran and the difficulties encountered in adapting to it:

*“The lifestyle in Iran is completely different*,* and I suffer from this every day. People in Iran have a unique lifestyle. In my opinion*,* it’s not good*,* but it’s unique. For example*,* shops close at around 9 PM*,* and when we go out at 9 PM*,* everything is closed*,* which is different from Iraq.”* (Interview 4).

*“Because I come from another country*,* there are customs and traditions here that I must adapt to. In Iran*,* each region has different behaviors that you don’t see in Iraq or other countries. So*,* I have to accept them and react based on that*,* because I am currently in Iran*,* and I must act like the people here. I respect the laws here.”* (Interview 7).

The extracts suggest that environmental factors such as unfamiliar food and housing conditions also contributed to the challenges faced by international students during their stay in Iran.

### Educational challenges

Participants identified *the lack of structured educational programs* and an *overload of academic content* as significant challenges facing international students. Several students expressed concerns regarding the demanding nature and extensive learning materials, particularly during their first semester. Additionally, the *professors’ limited English proficiency* and *ineffective teaching methods* posed considerable challenges for student. Other noted challenges included *insufficient exam scheduling* and *restricted study time*.

*“Professors can’t speak English well*,* and this makes it hard for us to understand the lessons.”* (Interview 4).

*“We get the exam schedule late*,* and when we get it*,* we don’t have enough time to study.”* (Interview 10).

These accounts highlight that language limitations among faculty members and organizational issues in the educational system created additional academic difficulties for international students.

### Cultural differences

The analysis of the findings revealed that one of the major issues faced by international students at IUMS is *cultural and ethnic differences*, which result in *difficulty in establishing social connections* with Iranian students and citizens. These cultural challenges were categorized into two main subcategories: *inappropriate behavior and cultural regulations at the university*, *and* a *lack of acceptance within society*.

Some students mentioned that inappropriate behavior from staff and faculty, along with strict of security measures at the university towards international students have been significant concerns:

*“One day*,* my shirt button was slightly open*,* and the security guard wouldn’t let me enter*,* while I saw many Iranian students in the same condition.”* (Interview 1).

Moreover, some students pointed out that certain members of host community, including some professors, exhibit discriminatory and unjust behavior towards individuals of non-Iranian nationality:

*“We feel that there is a difference between us and Iranian students. This is discrimination.”* (Interview 6).

Additionally, some international students mentioned experiencing humiliation, insults, and negative perceptions from the members of society as an additional challenge:

*“Yesterday in the supermarket*,* an Iranian guy told me about ‘oscola*,*’ which means fool or drunk*,* and this applies to all Iranians.”* (Interview 1).

The participants’ experiences reflect how cultural misunderstandings and differing social norms sometimes led to uncomfortable interactions with members of the host community.

### National attitudes

Two main subcategories related to nationalistic attitudes were identified: *discrimination in the enforcement disciplinary rules* and *discrimination and injustice in interpersonal behaviors*. Some participants reported that disciplinary regulations were applied more stringently to international students than to their Iranian counterparts. For example, female Iraqi students faced restrictions on entering the university due to their attire, while Iranian students faced no such issues:

*“When female students enter*,* the guards warn them about their clothes being too short. This is discrimination because no such issues exist for Iranian female students.”* (Interview 4).

Many international students expressed concerns about experiencing discriminatory or demeaning behavior from Iranian citizens. Specifically, their interactions often changed when individuals discovered that they were not Iranian:

*“Sometimes when people realize you’re not Iranian*,* their behavior changes and they stare at you.”* (Interview 3).

These statements indicate that perceived negative attitudes and strict social regulations occasionally influenced students’ sense of comfort and belonging in the host society.

### Personal issues

Another main issue for international students at IUMS is personal problems. Analysis of interview data indicates that a major contributing factor in this area is the lack of communication with family. Being distance from home and family, coupled with difficulties in establishing relationships within the host community, often leads international students to experience homesickness and feelings of estrangement:

*“As I mentioned*,* we are similar to Iranians*,* but it is difficult for us. We are far from our families. We don’t have a father*,* mother*,* brother*,* or relatives here. It’s just me and my sister here. It’s hard*,* but we’ve gotten used to it.”* (Interview 9).

*“We are far from our families*,* and this is hard for us. The only issue for me in this regard is that my friends and family are in Iraq*,* and I don’t have any other problem.”* (Interview 25).

The extracts demonstrate that homesickness and separation from family were common emotional challenges among international students.

### Financial issues

One of the main challenges confronting international students at IUMS pertains to financial issues, which can be classified into two subcategories: *financial problems* and *lack of basic banking facilities*. International students face high living expenses in Iran, including rent, food, transportation, and tuition fees. These costs have escalated significantly due to currency fluctuations. Many students face difficulties in transferring funds for tuition and living expenses. Complications arising from political and economic conditions in both their home country and Iran further exacerbate these challenges. Additionally, banking processes and currency conversion are time-consuming and risky due to security concerns:

*“Living expenses*,* rent*,* and tuition fees are much higher here compared to other countries.”* (Interview 7).

*“We cannot receive money directly from our families in Iraq. We have to use exchange offices*,* which is risky.”* (Interview 6).

These comments show that financial limitations and restricted work opportunities created additional pressure for some international students.

### Solutions for addressing the challenges of international students

Participants suggested potential solutions to the challenges they faced. These solutions are classified into six main areas: *educational* programs, *language courses*, *cultural awareness*, *personal resilience*, *financial* facilities, and *institutional supports*, as outlined in Table 2. The findings suggest that implementing some of these solutions could help mitigate existing barriers.

**Table 2:** Solutions for Addressing the Challenges of International Students

Main Category	Sub-category	Concepts
Educational Programs	Improving faculty communication skills	English-proficient faculty members selection
	Enhancing teaching and exams	Proper educational planning
Language Courses	Language training courses	Enhancement of English and Persian language proficiency
Cultural Awareness	Improving cultural awareness	Having experience in facing and dealing with insults from Iranian citizens
Personal Resilience	Personal adaptability	Increasing satisfaction through enhanced adaptability
	Social interaction	Increasing satisfaction through enhanced interactions with fellow native speakers
Financial Facilities	Cost management	Budget assignment and practicing cost-saving in various expenses
Institutional Supports	Dedicated support unit	Supporting the resolution of students’ daily problems

### Educational programs

Solutions suggested by the students for addressing the educational challenges can be categorized into two primary areas: *improving faculty communication skills* and “*enhancing the quality of teaching and examinations.*

The interviews revealed major issues, including communication gaps between students and professors, inadequate teaching by some faculty members, and the insufficient attention to the limited proficiency of international students in Persian. The university should prioritize selecting faculty members who are proficient in English rather than offering Persian courses:

*“I think the government and the university should pay more attention to the academic side*,* especially the part related to the language of doctors. You don’t know how difficult it is to understand what doctors are saying here.”* (Interview 4).

Furthermore, the university needs to improve the quality of teaching through effective educational planning. For example, professors should refine their teaching methods, tailor their instruction to students’ abilities, and provide additional explanations in class when necessary:

*“They should improve their teaching methods.”* (Interview 17).

To alleviate the demands of an intensive curriculum, practical measures can be implemented, such as using files prepared by professors instead of distributing extensive reading materials, reducing credit hours, or ensuring better coordination among course topics:

*“Don’t give us a big book like ‘Jancora*,*’ give us a PDF that the professor has prepared for us*,* and in my opinion*,* medical terminology 1 and 2 should be in the first semester*,* not in the second or third semester. Because what’s the point of taking histology and all of medical terminology and then taking medical language 1 and 2 afterward?”* (Interview 1).

The university should also provide the exam schedule earlier, ideally at the beginning of the semester, to help reduce mental stress and pressure during final exams. In consideration of students’ challenges with both Persian and English, it is recommended that the difficulty level of exams be adjusted. This change would facilitate greater comprehension of exam questions for international students rather than relying on complex wording:

*“Regarding exams*,* for example*,* Iranian students know their exam schedule from the beginning of the semester*,* but international students receive their exam schedule much later and have less time to study. When we get the exam schedule*,* we realize it is very difficult to study all the course material in two months. We need to prepare ahead of time. So*,* the exam schedule should be given to us from the beginning of the semester. We need to study before the exam.”* (Interview 10).

Participants emphasized that providing English-medium instruction and improving educational planning could significantly reduce many of the academic challenges they faced.

### Language courses

A potential solution to the challenges encountered by international students is to *enhance English and Persian language courses.* Several participants indicated that, due to the limited proficiency of most international students in these languages, it would be advantageous to offer remedial language courses for both languages prior to the commencement of university programs. Such initiatives would enhance academic progress and foster a deeper understanding of course content:

*“Consider that not everyone has good English. Therefore*,* if you can offer some introductory courses before the actual college program begins*,* it would be very helpful*,* and students won’t be shocked when they start the main course.”* (Interview 3).

*“For instance*,* to solve the Persian language problem*,* I could attend Persian language courses. If we want to learn Persian outside the university*,* it would take a year. But within the university*,* we can learn it in two or three months and teach others as well by organizing courses for them. If we have issues with English*,* we could also arrange English courses.”* (Interview 10).

The participants suggested that structured Persian language courses could facilitate communication and help international students adapt more easily to academic and social environments.

### Cultural awareness

*Improving cultural awareness* can serve as a strategy for addressing the challenges that international students encounter in their social interactions with the host community. In the light of issues such as discrimination, inappropriate national or racial behaviors, and mistreatment from certain individuals, a non-confrontational approach can facilitate the integration of students into the host society and promote their development as intercultural citizens. Strategies that promote tolerance of cultural differences, discourage racism, and encourage the dismissal of insults or potential discrimination can help mitigate further challenges:

*“If that person had spoken to me in Persian*,* I would have understood the insult*,* but I pretended I wasn’t interested in Persian. I acted normally because I didn’t want any problems to arise.”* (Interview 1).

*“I try to treat others well and act against racism to improve society.”* (Interview 24).

These suggestions highlight the importance of cultural orientation programs in promoting mutual understanding between international students and the host community.

### Personal resilience

We propose leveraging *the adaptability of individuals* as a solution to the challenges faced by students. Students often rely on their personal experiences, inner strength, and flexibility to overcome obstacles. By maintaining composure, valuing their unique personalities, and respecting the opinions and cultural differences of the host country’s citizens, individuals can enhance their satisfaction and foster better relationships with local citizens, thereby mitigating potential issues:

*“In fact*,* the first thing that is common among all students when facing problems is calmness. Everyone has their own unique personality and approach to dealing with problems. Some people are angry and aggressive*,* struggling to connect with their problems*,* making them even bigger. However*,* some others are balanced and try to solve their problems with reason*,* not confrontation and fighting. They use their intellect*,* talk it through*,* share ideas*,* and practice tolerance.”* (Interview 7).

By actively seeking friendships and increasing interactions with citizens of the host country and fellow nationals, international students can address problems such as isolation, homesickness, and feelings of loneliness:

*“Like I live with my friends*,* Iraqi friends or Arab friends or make events with my friends. Like this*,* yeah. Okay. Very good. Yes*,* that’s a help. Yes. Not to feel homesick. Help you to feel like a family. Uh-huh. Okay. We say when you’re in another country*,* you feel a little bit homesick. Yes*,* right.”* (Interview 6).

The participants’ experiences suggest that personal coping strategies and adaptability played an important role in overcoming many of the challenges they encountered.

### Financial facilities

Effective cost management can also help resolve specific financial challenges. By placing greater emphasis on expenses and implementing savings strategies in various areas, it is possible to manage some of the significant costs associated with the academic experience:

*“I make a list of expenses for each month and try to ensure that my spending doesn’t exceed that amount.”* (Interview 16).

Additionally, by sharing expenses with peers and fellow students, students can alleviate some of the high costs, such as housing rent:

*“No*,* the living expenses we didn’t have a problem. Because we live four persons in one home. Me*,* my sister*,* Sahra*,* Qaimi*,* the girls who gave a presentation with our friends. And one girl here in this university. But in eight or nine terms*,* we are four for Afghanistan. We live together and have a house. And everything we must live*,* we divide it to four.”* (Interview 9).

These statements emphasize that financial support and employment opportunities could significantly improve international students’ experiences.

### Institutional supports

Moreover, *establishing a specialized assistance or counseling center in Arabic or English at the university* could substantially assist in guiding and orienting students. This center would enhance students’ awareness of diverse administrative, academic, and cultural dimensions while addressing their specific concerns. Students proficient in the language could have a role in selecting the personnel responsible for this center or its independent section:

*“By assigning staff who speak Arabic*,* for example*,* in the counseling or assistant sections*,* this is very helpful for students who cannot speak English or Persian well.”* (Interview 3).

The extract illustrates the importance of institutional support systems in facilitating international students’ adjustment to university life.

### Opportunities of studying at IUMS

The numerous opportunities of studying at IUMS provide opportunities for students’ personal and academic growth. As displayed in Table 3, these opportunities are categorized into four main areas: *cultural*, *educational*, *institutional*, and *personal opportunities*, which are further explored in the following section.

**Table 3:** Opportunities of Studying at IUMS

Main Category	Sub-category	Concepts
Cultural	Cultural similarities	Cultural Affinity Between Iran and Neighboring Countries
	Ethics-based culture	A Positive Experience with the Attitude of People in the Residential Environment
	The culture, customs, and religion of Iran	A Sense of Satisfaction with the Cultural Environment, Traditions, and Religious Practices in Iran
Educational	Adequate educational quality	A Positive Attitude Toward Studying in Iran
Institutional	Library services	Satisfaction with the University Library
	Nutrition services	Satisfaction with the University Restaurant
	Support services	Access to Healthcare Services, Insurance, Recreational Activities, and Tuition Discounts
Personal	Satisfaction	Having a Positive Experience and Satisfaction with Living Conditions in Iran

### Cultural opportunities

*Cultural similarities* represent a significant advantage of studying at IUMS. These similarities with the host country foster mutual cultural understanding, which significantly influences the adjustment and comfort levels in a new environment:

*“Iran’s culture is similar to my country’s culture*,* it’s the same. I don’t have any problems in this regard.” (Interview 10)*.

An *ethics-based culture* identified in this research as one of the cultural advantages of studying at IUMS. Most Iranians demonstrate a strong cultural awareness and respect for diverse opinions and nationalities in their daily interactions. This characteristic significantly greatly facilitates the cultural adaption of international students and enhances their ability to engage comfortably with the local population:

*“I think it’s very different*,* much more positive. We always hear on TV or in the news that Iran is a very bad country. But when we come here to Iran*,* I realized that Iran has good people. The people here are very polite and kind to everyone around them. They don’t care about people’s nationality. I think TV or the news gives a very bad image of the people of Iran.”* (Interview 2).

The *culture*,* customs*,* and religion of people* offer significant cultural advantages for students studying at IUMS. Iran’s rich traditions of the community instill a sense of satisfaction and well-being among international students:

*“It hasn’t caused me any problems because every time I see that our culture is the same as yours—like the government*,* people*,* and language. Even the way we dress and the food we eat.”* (Interview 9).

The participants also highlighted positive cultural experiences that helped them become more familiar with Iranian traditions and social life.

### Educational opportunities

*Adequate educational quality* is a significant advantage in the educational process. The medical education system in Iran upholds rigorous academic standards, with IUMS being particularly distinguished across various educational, scientific, and research domains. Consequently, students can enhance their academic and professional skills by leveraging the university’s diverse educational and scientific resources, thereby gaining access to exceptional opportunities for academic advancement:

*“In fact*,* it is beyond my expectations. The educational conditions are better than in Afghanistan. This has been beyond my expectations because I don’t know if you are aware*,* but the educational level in Afghanistan is very low. I also attended university in Afghanistan and studied medicine for four semesters. I went to a Taliban university for four semesters*,* which was only for girls. We waited for six or seven months for the university to open*,* but it never did. We came here and saw the differences. Here*,* we started studying for the first time. As someone who has attended university in Afghanistan and compares it to here*,* I see many differences. In histology*,* we studied the different types of epithelium in various parts of the body. We studied this subject in Afghanistan as well*,* but we didn’t cover these topics. We only knew in theory how many types exist in the body*,* but when we came here*,* we saw all these types under the microscope. They showed us muscles*,* bones*,* and humans.”* (Interview 9).

*”For example*,* if we want to go to another university*,* the doctor doesn’t teach the most specific or important things that would benefit us. But the Iranian university provides that for me because I am trying. In my country*,* I took courses. I started studying medicine as a medical student in my country for one year*,* but here it is much more beneficial for me. It gives me the strength to be a good doctor in the future… After finishing seven years in Iran*,* I want to return to Iran and specialize here. I would prefer to specialize at Iran University of Medical Sciences. I don’t want to change my university. Iranian universities are very good.”* (Interview 10).

These accounts indicate that studying in Iran also provided valuable academic experiences and exposure to different educational approaches.

### Institutional opportunities

Participants reported library services as an organizational advantage of studying at IUMS. The quality of the university’s support programs for library services is commendable and meets acceptable standards:

*“Also*,* the library*,* which is the best library I have ever seen*,* is well-organized. I found it very useful for all students. These two main things really help me a lot.”* (Interview 2).

The second subcategory of institutional opportunities associated with studying at IUMS pertains to *nutrition services*. A notable feature of the university is its restaurant, which provides high-quality meals that contribute to student satisfaction:

*“The food is also very delicious. Iranian food is very tasty.”* (Interview 10).

*Support services* constitute another advantage of studying at IUMS. The university has implemented arrange of initiatives to assist international students, including recreational and sports programs, cultural activities, access to health insurance, tuition discounts, and more:

*“The university also takes us to picnics or paintball*,* which is really nice. The picnic is very good. For example*,* next week they are taking us to Opal.”* (Interview 9).

The extracts show that university facilities and services created several supportive opportunities for international students.

### Personal opportunities

The final subcategory identified, regarded as one of the advantages of attending the IUMS, pertains to personal opportunities. Several interviewees indicated that they derive a significant degree of personal fulfillment from their studies at this university. From a personal development perspective, they have acquired new skills and knowledge, and by familiarizing themselves with the cultural elements, customs, rituals, and traditions of the host society, they have enjoyed a gratifying and enriching experience:

*“In fact*,* it was an incredible experience for me because I made new friends from different cities and countries and became familiar with new traditions and customs. As you know*,* the geographical location here is difficult for us*,* and therefore*,* I learned many things here*,* which was very good.”* (Interview 7).

*“I had much worse expectations. I expected little support from the government. I expected to feel sad and depressed*,* but it is actually very good.”* (Interview 4).

Participants described personal growth, increased independence, and new social experiences as important outcomes of studying abroad.

#### The prospects of international students at IUMS

As presented in Table 4, the final category of results in this study examines the prospects of international students at IUMS. An analysis of the findings revealed that, from a personal perspective, returning to their home country and pursuing their education there was a primary objective for a significant number of students. The participants generally expressed that their preference was to purse their studies in their home country. Consequently, many conveyed a strong desire and enthusiasm to return to their homeland:

*“I hope I can continue my studies in Iran*,* but there are some differences compared to Iraq. However*,* my family is in Iraq*,* and they are my first priority. I can’t be away from them*,* and that’s the only thing that prevents me from staying in Iran.”* (Interview 2).

*”I will continue my medical studies in Iraq.”* (Interview 5).

**Table 4:** The prospects of international students at IUMS

Main Category	Sub-category	Concepts
Personal Prospect	Return to the Home Country	A strong desire to return home after graduation

These perspectives suggest that studying in Iran may contribute to students’ future academic and professional development.

## Discussion

This study contributes to the growing literature on international student experiences by providing qualitative insights into the challenges, solutions, and opportunities faced by international students at Iran University of Medical Sciences. By capturing participants’ perspectives, the findings offer a contextualized understanding of academic, cultural, and institutional factors shaping international students’ experiences in a non-Western educational setting.

To better interpret the findings, this study draws on the academic and sociocultural adaptation framework commonly used in research on international student experiences. This framework suggests that international students’ adjustment in host universities is shaped by multiple interconnected dimensions, including academic adaptation, sociocultural adjustment, personal and psychological well-being, and institutional support. Organizing the discussion around these dimensions allows for a more systematic understanding of the challenges and opportunities experienced by international students at Iran University of Medical Sciences (IUMS).

Research consistently shows that the presence of challenges among international students is a common and intrinsic aspect of higher education. Living and studying in an unfamiliar social and academic culture exposes these students to various difficulties, including loneliness, homesickness, financial stress, issues with food and accommodation, language barriers, challenges in understanding lectures, adapting to social norms, and interacting with individuals from diverse cultural backgrounds. These issues warrant further examination and support [27]. Among these challenges, language barriers are identified as one of the most significant and obstacles to academic learning and social interaction for international students [28]. Insufficient proficiency in the host country’s language often serves as a major source of stress and can lead to numerous academic and social difficulties [29].

From the perspective of academic adaptation, language barriers and educational structures emerged as key challenges for international students at IUMS. The findings of this study also revealed that language barriers are among the most critical challenges faced by international students at IUMS. 51.6% of participants reported limited proficiency in both Persian and English. Due to their inadequate command of Persian, international students experienced difficulties in both academic and non-academic interactions with Persian-speaking peers and instructors. Additionally, insufficient English skills impeded their ability to comprehend course content and communicate effectively. Conversely, the limited English proficiency of some Iranian students also adversely affected collaborative learning and knowledge exchange with their international peers. These findings are consistent with previous studies that have identified language-related challenges as a major obstacle for international students [28–34]. Many studies have highlighted that insufficient proficiency in the host country’s local or commonly spoken language poses a significant challenge for international students [29–31].

In alignment with the current research, other studies have underscored the limited proficiency in Persian language among international students. Tajvar et al. [32] identified language barriers as a significant challenge for international students at Iranian universities noting that difficulties in understanding Persian language and course content negatively affect students’ academic performance and class participation. Similarly, Nakhoda et al. [28] mentioned the limited proficiency in Persian, as a primary factor affecting the satisfaction of international medical students in Tehran, as it impeded their ability to understand educational materials. Furthermore, research by Kheradmand et al. [34] and Jamshidi et al. [29] has also identified language deficiency as one of the most pressing challenges faced by international students.

In terms of sociocultural adaptation, international students reported difficulties related to differences in social lifestyles, cultural norms, and interactions with members of the host community. Additionally, the present study identified by international environmental issues as a challenge encountered by international students, particularly dissatisfaction with the differences in social lifestyles and difficulties associated with adapting to them in Iran. Correspondingly, several prior studies have documented environmental challenges, such as social isolation and obstacles related to socio-cultural adjustment within the host environment [27, 31, 35].

Educational programs were also identified as a significant issue for international students in this study. The lack of well-structured academic programs designed specifically for international students, coupled with heavy, complex, and at times overwhelming course materials, were found to hinder learning and academic performance. This finding aligns with previous research; for instance, Jamshidi et al. [29] reported that the high volume of specialized coursework posed a significant challenge for international students.

Another educational issue identified in this study was the inconsistency in academic laws and regulations, which included poorly scheduled examinations, insufficient study time during exam periods, and delayed communication absorbing important information. These factors adversely affect the quality and effectiveness of academic processes. Findings from related studies further corroborate the results of the present research. Nakhoda et al. [28] reported that demanding, poorly structured, and intensive academic programs were a substantial source of psychological pressure and stress for international students. Similarly, Tajvar et al. [32] identified that coping with rigorous academic schedules was one of the key challenges faced by international students in Iranian universities. These findings reinforce the conclusions drawn in the current study.

Globally, the quality of interactions among faculty, students, and university staff is considered a critical factor influencing the satisfaction of international students. These students often seek meaningful friendships and social engagement with local peers; when such expectations are unmet, they may experience disappointment or social isolation [28].

Consistent with the present study, previous studies have also emphasized the impact of cultural differences and reported instances of inappropriate behavior exhibited by faculty members, students, and local citizens toward international students [27–29, 32]. In a related study, Tajvar et al. [32] identified cultural challenges as a significant concern for international students. They specifically, noted that pronounced cultural differences—such as strict dress codes, social norms, and religious practices—may diverge from those familiar to international students, potentially leading to feelings of isolation or confusion.

This study also identified nationality-based attitudes as an additional dimension of the challenges faced by international students. The findings revealed that discrimination and inequitable treatment from certain faculty members, as well as inconsistencies in the enforcement of specific university rules and disciplinary regulations between Iranian and international students, along with overtly racist and offensive behaviors from some members of the host community, have contributed to negative experiences for international students. Several previous studies have also confirmed the existence of discrimination between international students and the host community, aligning with the findings of the present research [28, 30].

From a personal adjustment perspective, several participants described emotional challenges related to living away from their home country and family. Another finding from the interviews regarding the challenges faced by international students pertained to personal issues. The study’s results indicated that a lack of contact with relatives, separation from one’s homeland and family, and the inability to establish connections with the host community place international students in psychologically challenging situations, leading to feelings of alienation, loneliness, and homesickness. In a similar study, Soltani et al. [27] reported that a significant challenge faced by international students is the sense of separation from their home country, compounded by difficulties in communicating with the local community and fellow students, which exacerbates their feelings of homesickness. Furthermore another study emphasized the stressful and discouraging living conditions of international students, which raise from unfamiliarity with the new environment and the distance from their home country [28].

In this study, financial challenges emerged as another issue for international students, with financial problems being one of the primary concerns. It was found that the cost of living and studying in Iran—including rent, accommodation, utilities (water and electricity), food, transportation, and tuition fees—is relatively high and burdensome. Moreover, students faced additional another financial challenges related to obtaining or converting foreign currency at exchange offices for university tuition payments, a process that is often a time-consuming, due to political conditions. Several previous studies corroborate the findings of the present research, highlighting the financial problems experienced by international students [27, 28, 31–33].

Tajvar et al. [32] demonstrated in their study that financial constraints—such as high tuition fees and elevated costs of living in Iran, particularly housing and food—pose significant challenges for international students. Similarly, studies conducted by Hamzeh et al. [31] and Bashzar et al. [33] reported financial difficulties and the burden of educational expenses experienced by international students. Nakhooda et al. [28] highlighted various aspects of financial hardship, including difficulties in obtaining foreign currency, exchange rate fluctuations, and the rising cost of currency conversion, which collectively hinder students’ ability to afford tuition fees. In a related study, Soltani et al. [27] found that international students face multiple financial challenges, particularly due to the increasing costs of living and studying, including housing and rent.

Institutional support represents another important dimension of international student adaptation within the host university. The second category of findings indicated that implementing educational programs, language courses, cultural awareness, personal resilience, financial facilities, and institutional supports can help mitigate some of the existing barriers and subsequently improve the academic experience of international students at IUMS.

The findings revealed that critical educational strategy to address these challenges is to eliminate instruction in Persian and employ faculty proficient in English. This approach can reduce communication barriers between instructors and students. Similarly, Tajvar et al. [32] emphasized the importance of teaching in languages other than Persian as a means to alleviate some of the academic challenges faced by students. Another identified educational strategy to enhance teaching quality involves adjusting rigorous and intensive curricula, as well as the difficulty level of examinations. This includes better planning in teaching practices, reducing course credits, ensuring thematic and academic coherence among courses, and providing exam schedules at the beginning of the semester, allowing sufficient time for students to prepare.

A significant finding in the area of overcoming challenges pertains to the use of language-based strategies. A critical measure was the implementation and enhancement of English and Persian language training programs for international students, aimed at strengthening their communication skills and academic capabilities. As Hajar suggests [36], individuals act more purposefully when they recognize the value of specific activities in overcoming environmental limitations and achieving their goals. In this study, students’ awareness of their language weaknesses motivated them to focus on improving their English and Persian language proficiency as a means to achieve academic success. Some previous studies also support the findings of the present research regarding language-related strategies. For instance, Tajvar et al. [32] emphasized the importance of institutional support in providing Persian language classes to international students. Similarly, Hamzeh et al. [31] highlighted the effectiveness of targeted language instruction in overcoming language challenges, particularly in understanding unfamiliar medical terminology and related concepts.

Another strategy identified in this study to address the challenges faced by international students involved the adoption of non-confrontational approaches. The findings indicated that promoting cultural awareness and mutual understanding in interactions with members of the host society—particularly when encountering discriminatory or racially insensitive behaviors—can help alleviate some of these issues. Employing non-aggressive responses, such as tolerating cultural misunderstandings, disregarding offensive remarks or negative stereotypes, and avoiding nationalistic bias, may facilitate cultural integration, foster intercultural growth, and contribute to students’ identity development. Findings from previous studies also support this aspect of the current research. For example, Hajar et al. [36] reported that some international students improved their interactions with the host community and experienced greater comfort in multicultural environments by demonstrating tolerance toward cultural differences and respecting diverse perspectives. Hamzeh et al. [31] suggested that a practical approach to managing personal challenges—such as stress, burnout, fatigue, and emotional pressure—among international medical graduates is resilience, defined as the use of an individual’s internal adaptive capacity to cope with stressful situations.

One strategy identified in this study was financial coping. The findings indicated that managing expenses through cost-saving measures and sharing expenses with peers can help alleviate some of the financial burdens faced by international students.

Another organizational strategy identified in this study was the establishment of a dedicated support or advisory center within the university, staffed with personnel proficient in Arabic or English. Such a center could assist international students in expressing their needs and concerns while also providing guidance and support in administrative, educational, and cultural matters. Several previous studies have corroborated the solutions proposed by the interviewees. Tajvar et al. [32] noted that one effective way to address the challenges faced by international students is for the host university to provide academic counseling services. Similarly, Soltani et al. [27] reported the necessity for universities to establish a counseling center aimed at guiding students in various areas, such as administrative, educational, and related academic matters.

The third category of findings revealed that the cultural, institutional, academic, and personal opportunities of studying at the international branch of IUMS could significantly contribute to students’ academic and personal development. A key advantage identified was the cultural affinity and similarities between international students and the host society, which markedly enhanced their adaptation and comfort. Additionally, the ethics-based culture of the host community—characterized by mutual cultural understanding and respect for diverse nationalities—facilitated more effective and comfortable interactions with international students. Moreover, the richness of Iranian culture and traditions was perceived by many students as a positive and gratifying aspect of their experience.

In this study, institutional opportunities were also identified as a positive aspect of studying at the international branch of IUMS. The findings indicated that the university’s support programs—such as access to library services, meal plans and nutritional support, recreational and cultural activities, health insurance coverage, tuition discounts, and other supportive measures—were generally of satisfactory quality and contributed to overall student satisfaction.

Another benefit of studying at the international branch of IUMS identified in this study was the high quality of education and academic standards within the university’s medical education system. This advantage has provided international students with valuable opportunities for academic growth and the development of professional skills.

Furthermore, interviews revealed personal opportunities experienced by students. Exposure to the host community’s culture, customs, and traditions enabled international students to gain new knowledge and positive personal experiences, leading to increased personal satisfaction. These findings align with previous research; for instance, Tajvar et al. [32] demonstrated that international students had numerous opportunities for personal and academic growth, cultural immersion, and skills acquiring.

In the fourth category of findings, participants articulated their future outlook. The results indicated that, from a personal perspective, 41.93% international students preferred to return to their home countries after completing their studies rather than staying in the host country. This aspect of the study is supported by previous research; for instance, Hajar et al. [30] found that none of the international students expressed desire to stay in the host country after graduation.

Overall, this study contributes to the existing literature by providing qualitative insights into the experiences of international students in a non-Western educational context. While many previous studies have focused on Western universities, this research highlights how academic, sociocultural, personal, and institutional factors interact to shape the experiences of international students in an Iranian medical university. By organizing the findings through an academic and sociocultural adaptation perspective, this study offers a more comprehensive understanding of the challenges and opportunities faced by international students and provides practical implications for improving institutional support and educational policies.

### Limitaions and future research

Despite providing valuable insights into the experiences of international students at Iran University of Medical Sciences, this study has several limitations that should be acknowledged. First, the study was conducted within a single university context, which may limit the generalizability of the findings to other institutions or educational settings. Second, the sample size was relatively limited and consisted only of international students studying at IUMS; therefore, the perspectives of other stakeholders, such as faculty members or university administrators, were not included. Additionally, as this study employed a qualitative design, the findings reflect the participants’ subjective experiences and may not represent the experiences of all international students.

Future research could address these limitations by including participants from multiple universities and diverse educational contexts. Quantitative or mixed-methods studies may also help provide broader evidence regarding the challenges and adaptation processes of international students. Furthermore, future studies could explore the perspectives of faculty members, administrative staff, and policymakers to gain a more comprehensive understanding of the factors influencing international students’ experiences and support systems in host universities.

## Conclusion

This study revealed a range of challenges faced by the international students at IUMS, encompassing language, environmental, educational, cultural, nationality-based, personal, and financial issues, many of which hinder their learning processes and academic success. The findings also underscore the importance of implementing practical solutions to mitigate these barriers, including educational, language, cultural awareness, personal, financial, and institutional strategies. Despite these challenges, the study found that international students have capitalized on various opportunities—such as cultural, institutional, academic, and personal advantages—to achieve significant academic and personal growth. The insights generated from this study are invaluable for university administrators, academic leaders, and higher education policymakers, particularly in shaping both short- and long-term strategies related to the recruitment and support of international students. These results help stakeholders gain a better understanding of the current status of international students. Consequently, it is recommended that universities engage in regular monitoring and pursue further research in this domain as part of their operational planning. While the findings can be cautiously generalized to international students in other medical universities across the country, it is advisable that similar studies be conducted at other institutions. Comparative and analytical research will contribute to a deeper and more comprehensive understanding of the challenges faced by international students.

## Supplementary Information

Supplementary Material 1.

Supplementary Material 2.

Supplementary Material 3.

## Data Availability

The anonymized interview transcripts and coded data that support the findings of this study are available from the corresponding author upon reasonable request.

## Abbreviations

IUMS: Iran University of Medical Sciences
HE: Higher Education
