# Supplementary material for: Empowering international students at iran university of medical sciences: challenges, solutions, and opportunities
Source: BMC Med Educ. 2026 May 28;26:1212. doi: 10.1186/s12909-026-09493-0 (PMC13404166; doi:10.1186/s12909-026-09493-0)
Supplement: Supplementary file 2 — Supplementary Material 2. [file 12909_2026_9493_MOESM2_ESM.pdf]

Iran University of Medical Sciences

Research Ethics Committees Certificate

Approval ID:	IR.IUMS.REC.1403.226	Approval Date:	2024-06-02
Evaluated by:	Research Ethics Committees of Iran University of Medical Sciences		
Status:	Approved		
Approval Statement:	The project was found to be in accordance to the ethical principles and the national norms and standards for conducting Medical Research in Iran.<br>Notice:<br>1. Although the proposal has been approved by the Biomedical Research Ethics Committee, meeting the professional and legal requirements is the sole responsibility of the PI and other project collaborators.<br>2. This certificate is reliant on the proposal/documents received by this committee on 2024-06-02. The committee must be notified by the PI as soon as the proposal/documents are modified.		
Proposal Title:	Investigating the challenges for international students at Iran University of Medical Sciences and providing solutions		
Principal Investigator:	Name: Rafat Bagherzadeh<br>Email: bagherzadeh.r@iums.ac.ir		

Dr. Abdolreza Pazouki
Committee Director
Iran University of Medical Sciences

Dr. Reza Falak
Committee Secretary
Iran University of Medical Sciences
