# Supplementary material for: Empowering international students at iran university of medical sciences: challenges, solutions, and opportunities
Source: BMC Med Educ. 2026 May 28;26:1212. doi: 10.1186/s12909-026-09493-0 (PMC13404166; doi:10.1186/s12909-026-09493-0)
Supplement: Supplementary file 3 — Supplementary Material 3. [file 12909_2026_9493_MOESM3_ESM.pdf]

بسمه تعالی

تاریخ: ۱۴۰۴/۱۲/۰۳

"گواهی شرکت در طرح"

بدینوسیله گواهی می شود؛ سرکار خانم/جناب آقای **شیوامالگرد** در **طرح اچ اس آر** با مشخصات ذیل فعالیت پژوهشی داشته اند.

عنوان طرح : "**بررسی مشکلات دانشجویان بین الملل دانشگاه علوم پزشکی ایران و ارائه راهکار** "

کد طرح: **27602-52-8-1402** شروع طرح: **1402/10/18** پایان طرح: **1403/08/18**

وضعیت : **خاتمه یافته** تاریخ تصویب **۱۴۰۲/۱۰/۱۸** سمت: **همکار دوم**

افراد شرکت کننده درطرح: **۵ نفر (رفعت باقرزاده- لیلا نعمتی انارکی- مطهره آقاشاهی- افسانه دهناد- شیوا مالگرد)**

مبلغ قرارداد **۵۴۵،۵۰۰،۰۰۰**

کارفرما : **معاونت پژوهشی دانشگاه علوم پزشکی ایران**

این گواهی بنا به درخواست نامبرده صادر گردیده است و فاقد ارزش دیگری می باشد.

معاون تحقیقات و فناوری دانشگاه

دکتر مجید صفا

آدرس – تهران بزرگراه شهید همت غرب بین تقاطع شیخ فضل ا... نوری و شهید چمران- تلفن مرکز 86701 - فکس 88622703 - تلفن 86702503
